# Fate

**Published:** 1902-03

**Authors:** Susan Marr Spaulding


### Fate.

Two shall be born, the whole wide world apart,
And speak in different tongues, and have no thought
Each of the others being, and no heed;
And these o'er unknown seas to unknown lands shall cross,
Escaping wreck, defying death;
And all unconsciously shape every act,
And bend each wondering step to this one end:
That one day, out of darkness they shall meet,
And read life's meaning in each others eyes.

And two shall walk some narrow way of life,
So nearly side by side, that should one turn
Ever so little, left or right,
They needs must stand acknowledged, face to face.
And yet with wistful eyes that never meet;
With groping hands that never clasp, and lips
Calling in vain to ears that never hear,
They seek each other all their weary days,
And die unsatisfied. And this is Fate.

—*Susan Marr Spaulding.*

